# Ethical considerations in utilizing artificial intelligence for analyzing the NHGRI’s History of Genomics and Human Genome Project archives

**DOI:** 10.7191/jeslib.811

**Published:** 2024-03-05

**Authors:** Mohammad Hosseini, Spencer Hong, Kristi Holmes, Kris Wetterstrand, Christopher Donohue, Luis A. Nunes Amaral, Thomas Stoeger

**Affiliations:** Northwestern University Feinberg School of Medicine, Chicago, IL, USA; National Institutes of Health, Bethesda, MD, USA; Northwestern University Feinberg School of Medicine, Chicago, IL, USA; National Institutes of Health, Bethesda, MD, USA; National Institutes of Health, Bethesda, MD, USA; Northwestern University, Evanston, IL, USA; Northwestern University Feinberg School of Medicine, Chicago, IL, USA

**Keywords:** artificial intelligence, AI, large language models, National Human Genome Research Institute (U.S.), Human Genome Project, ethics, responsibility, privacy

## Abstract

Understanding “how to optimize the production of scientific knowledge” is paramount to those who support scientific research—funders as well as research institutions—to the communities served, and to researchers. Structured archives can help all involved to learn what decisions and processes help or hinder the production of new knowledge. Using artificial intelligence (AI) and large language models (LLMs), we recently created the first structured digital representation of the historic archives of the National Human Genome Research Institute (NHGRI), part of the National Institutes of Health. This work yielded a digital knowledge base of entities, topics, and documents that can be used to probe the inner workings of the Human Genome Project, a massive international public-private effort to sequence the human genome, and several of its offshoots like The Cancer Genome Atlas (TCGA) and the Encyclopedia of DNA Elements (ENCODE). The resulting knowledge base will be instrumental in understanding not only how the Human Genome Project and genomics research developed collaboratively, but also how scientific goals come to be formulated and evolve. Given the diverse and rich data used in this project, we evaluated the ethical implications of employing AI and LLMs to process and analyze this valuable archive. As the first computational investigation of the internal archives of a massive collaborative project with multiple funders and institutions, this study will inform future efforts to conduct similar investigations while also considering and minimizing ethical challenges. Our methodology and risk-mitigating measures could also inform future initiatives in developing standards for project planning, policymaking, enhancing transparency, and ensuring ethical utilization of artificial intelligence technologies and large language models in archive exploration.

## Summary

The National Center for Human Genome Research, the precursor to the National Human Genome Research Institute (NHGRI), was created at the National Institutes of Health (NIH) in 1989 to guide the US development of the Human Genome Project (HGP), a watershed moment in biomedical research. Recognizing the historic value of the HGP, NHGRI preserved and archived a significantly large number of internal documents of the HGP and subsequent genomics initiatives. Presently, this archive, which is the only historic genomics and Human Genome Project archive within NIH, houses an estimated two million pages that include cost-benefit analyses, interim reports, grantee presentations, internal memos, strategy papers, internal working documents, server logs, presentation, emails and scanned letters among senior personnel and toward external key stakeholders, and more—essentially anything that has been produced in relation to the institute’s core mission guiding and funding genomics.

**Figure 1:**
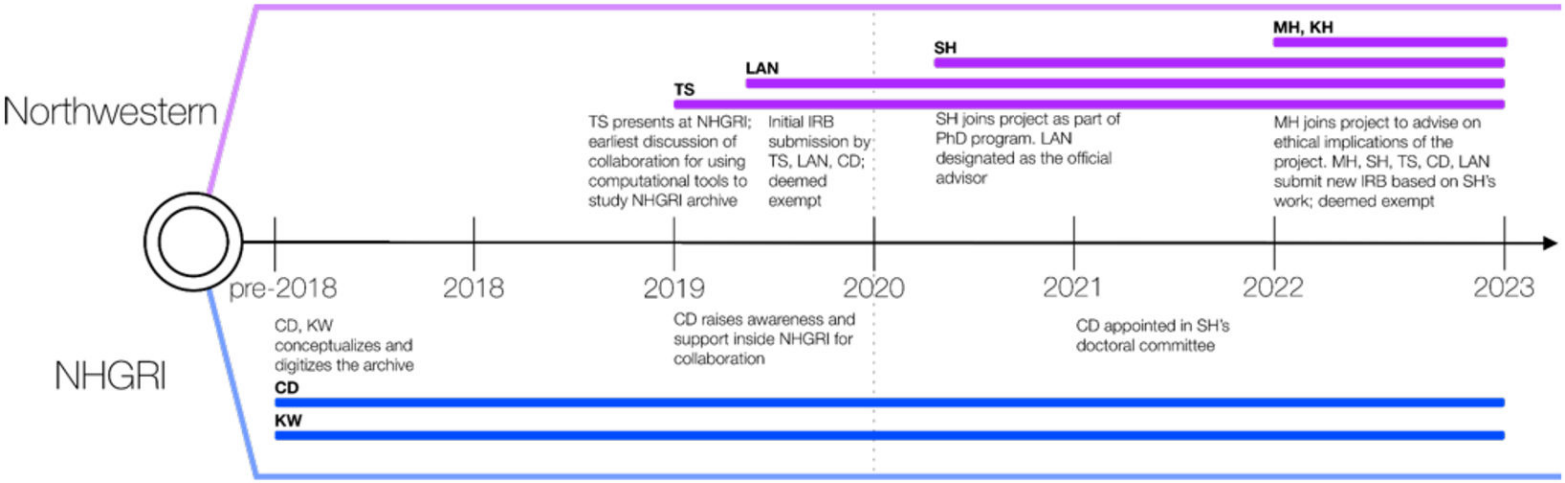
Collaboration timeline between the History of Genomics program of National Human Genome Research Institute (NHGRI), Northwestern University’s Amaral Lab and Galter Health Sciences Library.

A collaboration between Northwestern University’s Amaral Lab, Stoeger Lab, Galter Health Sciences Library, and the History of Genomics Program at the NHGRI now aims to computationally analyze and understand the decision-making processes behind a massive international public-private biomedical project (see figure 1). The origins and importance of the data motivated us to be especially cautious of ethical considerations regarding opening and studying this rich archive with tools powered by artificial intelligence (AI). Our observations could help members of the GLAM (Galleries, Libraries, Archives, and Museums) community navigate the numerous ethical challenges of using AI and large language models (LLMs) when processing and exploring archives. Future efforts could gain insights from our approach to establish best practices for project planning, policy-making, fostering transparency, and promoting responsible use of AI when exploring archives.

## Project development

### Human Genome Project (HGP)

The initial sequencing and analysis of a human genome has been described as a transformative moment in the history of biological research (Hood and Rowen 2013). Knowing the sequence of nucleic acids within human DNA promised to accelerate the identification of human genes. Knowledge about the human genome’s sequence, enabled genomics researchers to monitor within a single experiment a near-complete set of genes, rather than studying them *individually*. This helped to better characterize the genetic architecture of health and disease, as well as common and rare genetic conditions, yielded insights into the origins and history of our species, and enabled novel types of studies. Since then a significant number of polymorphisms within genes has been discovered, and their biological and medical significance evaluated. The initial sequencing of the human genome was achieved through two competing efforts. An international consortium, called the International Human Genome Sequencing Consortium (IHGSC), and a commercial effort, spearheaded by Celera Genomics, with the latter initially striving to patent the genes discovered during their sequencing efforts.

On June 26th, 2000, members of both initiatives, together with national and international politicians publicly announced the initial completion of their efforts in a press conference at the United States White House, where the former president, Bill Clinton, noted “we are here to celebrate the completion of the first survey of the entire human genome. Without a doubt, this is the most important, most wondrous map ever produced by humankind” (“June 2000 White House Event” 2012). Simultaneously, Clinton also pointed out the inherent ethical considerations that surround research into the human genome: “… increasing knowledge of the human genome must never change the basic belief on which our ethics, our government, our society are founded.”

### Project’s scope and foundational phase

Our project has been exploring the history of the HGP directed by the NHGRI between c. 1993 and c. 2008. During this period scientists achieved transformative advances in mapping and sequencing technology, as well as significant developments in organismal and comparative sequencing, disease gene mapping, and insights into the genetic architecture of health and disease. This period also included the completion of various iterations of the genome sequence and the development of sundry genomics programs. Examples included the publication of the initial draft sequencing results in 2001, the formal conclusion of the project in 2003, and the development of landmark genomics programs such as the International HapMap Project, which developed a genome wide comparative map of human variation (“International HapMap Project” 2012). In 2012, Eric Green, who had been appointed NHGRI Director in 2009, proposed a unique History Program at the NHGRI to not only preserve and analyze these materials, but to develop an international effort to promote scholarship into the history of the HGP using these materials. Led by Dr. Chistopher Donohue and Kris Wetterstrand, MS, acting as the liaison to the Division of Extramural Research, the History of Genomics Program continues to preserve the history of the HGP and promote research into its rich history.

A major step for preservation of the records was the digitization of the day-by-day programmatic aspects of HGP guidance and funding, which later moved to the preservation of shared documents and communications on follow-up efforts to the HGP after 2003. The vast majority of the hard-copy materials were digitized via bulk scanning methods, using 300 DPI, Panasonic High-Speed Scanner for bulk scanning. Some materials, particularly those related to the development of sequencing technology, were digitized using “hand scanning” at a much higher resolution. Most, if not all, of the computationally investigated materials, are the product of bulk scanning or are “born digital” resources such as those created using Microsoft Word. Though these preservation efforts were primarily directed toward the NIH and NHGRI, the archive also has limited representation from other organizations involved in the project (e.g. the Department of Energy, Celera Genomics).

The motivation to implement the structured digitization of the materials in collaboration with scientists outside of the NIH grew from the specific shared interests and competencies of those involved. The current project had its birth in February 2019, when Dr. Christopher Donohue invited Dr. Thomas Stoeger to present a guest lecture at the NHGRI’s History of Genomics and Molecular Biology Lecture Series to speak about his quantitative research into the natural and social factors that underlie research into individual human genes. Follow up discussions created an opportunity for collaboration and to extend computational approaches for studying scientific literature toward archival documents surrounding the HGP.

### Technical and regulatory requirements

Over the subsequent months, Drs. Donohue and Stoeger used a set of ~1,000 documents to develop an initial project proposal and outline, and piloted feasibility studies. The goal of pilot studies was to show prospects of this endeavor and gain support for a larger effort within the NHGRI. A key insight was that the project needed relatively little computing power, and that a local workstation could be used to avoid the risk of potential security breaches that could occur in a decentralized infrastructure. Furthermore, it was hypothesized that tools that had been developed in other scientific contexts such as Gensim (for automated keyword extraction) and doc2vec (to organize documents by similarity) could be reused for some of the necessary tasks.

Importantly, it did not escape their attention that the data contained instances of potentially sensitive information (such as potential interventions in clinical genetics and broad discussions of health-related questions) that could possibly be retrieved through very specific queries. One solution was to limit the possibility of malicious queries by conceptually separating data preprocessing and data analysis. Accordingly, different redaction procedures such as masking sensitive documents, people names, and timestamps were implemented. Furthermore, guidelines that allowed the NHGRI to forbid certain usage scenarios were refined in a later stage of the project.

This initial technical proof and the resulting design of the computational- and data-workflow were essential to create demos and gain support for the project within the NHGRI. Later, an application was submitted to the Institutional Review Board (IRB) at Northwestern University for the development of a content extraction platform. The project was deemed low-risk and thus exempt.

### Scale up phase

In order for the project to progress, it became necessary to have someone dedicate their full attention to it, something that neither Dr. Donohue nor Dr. Stoeger could afford. Fortunately, an incoming graduate student in the Amaral lab and chemical and biological engineering graduate program at Northwestern University—Spencer Hong—was interested in spearheading the research.

The first step in the scaling up of the project was to go from ~1,000 initial documents to ~20,000 (corresponding to 3,894 files), which had been frequently accessed or requested by historians of science. This “core” sample served as a valuable and well-reviewed corpus that is representative of document types and scientific projects in the entire archive, which we estimate to be around 2 million pages.

The scaling up created several challenges. On the technical level, we had to create novel algorithms and approaches for content extraction that were outside of the predicted scope of the project. For instance, models for scan segmentation (a task where multi-document scans are separated into their logical boundaries) did not exist at the start of the study, and only recently did LLMs become widely available and enabled us to create sufficient high quality synthetic data for model training.

Training deep learning models required large computer resources that normally would be provided by an institutional cluster (e.g. Quest at Northwestern) or cloud services (e.g. AWS). However, the data use agreement between Northwestern and NHGRI mandated that the data stay isolated in a local, encrypted drive. Therefore, the Northwestern team received an in-equipment grant from NVIDIA that supplied two large GPUs, each with enough memory to train large language models. A dedicated workstation was built with encrypted drives and a fail-safe backup; with no other projects or users added to the workstation, we ensure privacy and protection for the NHGRI archive data. This workstation houses all of the analyses and models created in the project. Instead of relying on cloud services, we used software alternatives and models that could be set up locally to avoid compromising data privacy.

When drawing from improvements in external algorithms and computational tools, one discrepancy remains. Specifically, in preservatory contexts, metadata of historical archives of federal agencies may need to follow additional metadata standards defined by other parties such as the U.S. National Archives and Records Administration (NARA) metadata requirements (TransAccess 2019). This issue was pointed out by Zachary Utz, MA, Archivist and Public Historian, based at the NHGRI History of Genomics Program at the NIH. While it is possible to implement such standards, we have not yet done so because we were concerned that an early focus on these requirements may discourage a greater exploration of computational approaches to extract metadata. We anticipate that later stages of the project will involve following specific NARA requirements and maturity of the project will be determined by adherence to such standards.

The Galter Health Sciences Library at Northwestern University’s Feinberg School of Medicine has played a unique role in this project by providing guidance on how to manage the data in light of the new NIH data management and sharing policies (Gonzales et al. 2022; Hughes et al. 2023), and in anticipating and avoiding ethical issues that are common in social science projects that involve using large digitized datasets (Hosseini et al. 2022). One conceptual challenge was how to best include ethics as an integral part of the project rather than an afterthought. At this point Dr. Hosseini, an ethicist at Northwestern’s Department of Preventive Medicine and based at Galter Library, offered assistance to develop a plan to conduct the study in accordance with ethical norms and best practices.

## Background

The primary reason for digitization was to improve the accessibility of the archive, and to facilitate subsequent studies. Potential applications could range from identifying documents of interest to historians, to allowing large contextual analyses that would be painstakingly manual and labor intensive for close reading. Future explorations could investigate specific aspects of the HGP (e.g. how different centers were led, the degree to which governance and innovation of the project changed over time), or use the HGP as a model for understanding contemporary history of science and institutional decision making.

Some of the technical challenges we faced in this project arose from the lack of preceding studies with similar characteristics, which required developing novel tools and building connections between existing tools. Moreover, while the sample corpus is large in terms of the manual effort required for its reading, it is nonetheless too small for the training of cutting-edge machine learning approaches. We solved this challenge by adopting the use of synthetic data, which additionally freed us from concerns about privacy, scale, or data diversity. For this purpose and depending on the task, we used synthetically arranged document boundaries, superimpositions of handwriting, and other features observed in the corpus.

At a non-technical level, a lack of precedents also required developing guidelines and procedures that will respect the interests of involved parties and the privacy of individuals mentioned in the archive. Though individuals’ identity is usually known to historians of science, computational data mining could, for instance, reveal potentially private or stigmatizing information and it was our ambition to prevent these instances. We started with existing tools such as Gensim (a Python library for analyses related to documents) and spaCy (open-source software library for advanced natural language processing) for content extraction. These tools generally worked well but required data preprocessing to remove handwriting. Additionally, we developed customized machine learning annotation tools to review models and refine them. For guidance and inspiration, we also considered the ArchivesSpace platform to create an interlinked database of metadata and documents, and de-identification tools of Amazon Web Service to mask sensitive information and prevent transfer to another storage location.

## Ethical considerations

### Breach of confidentiality and harming subjects and/or their reputation

The availability of large-scale, machine-readable data enables the successful implementation of queries that could retrieve private or sensitive information and harm individuals captured in such data. Experts have in the past explored and discussed this issue using specially trained, fine-tuned models (Ahmed et al. 2021; Meystre et al. 2010). However, complete de-identification remains a challenging issue, one that has become more fraught with AI advancement and increased data access. User data, even when de-identified, have been shown to be re-identifiable (Barbaro and Zeller Jr. 2006; Narayanan and Shmatikov 2008) and even aggregate statistics about a dataset can undermine de-identification efforts (Dick et al. 2023). We, as the scholars wishing to computationally study documents that capture numerous individuals, wished to avoid a scenario where we inaccurately state that our data was fully de-identified, when it realistically was not.

Our data origins (from early 1990s to the late 2000’s) and content (informal and formal correspondences, handwritten notes, receipts, and other paperwork that would exist in any large organization) introduce colloquial, conversational, and informal language that individuals may or may not choose to be associated with. Documents like handwritten notes, email correspondence about non-scientific topics, and recorded personal communications may contain information that could bring possible personal and social harms to subjects captured in the archive.

### The particular risks associated with handwriting

We can reasonably anticipate that titles, handwriting, and timestamps may all risk reidentification. Handwriting specifically, may facilitate reidentification through text as well as handwriting style. Indeed, handwriting has been proven to be unique to individuals (Faundez-Zanuy et al. 2020), and the scale of the archive would be enough for a machine to trace back the handwriting style to an individual. Furthermore, handwritten text still often escapes traditional engines, which means that the Protected Health Information (PHI) included in handwritten text do not get flagged by existing PHI detection models. The nature of handwritten text indicates a colloquial, or informal, usage of language leading to writing more specific to the individual than professional use of language in the workplace. Lastly, because of the abundance of email correspondences in the archive, the timestamps could generate approximate chains of existing text, which may create longer threads of text than existing standalone text that can help reidentify individuals.

For these reasons, the possibility of complete de-identification of the archive may be infeasible and undesirable as it could result in uncontrolled reuse. For instance, declaring a dataset *de-identified* might make it suitable for the use of AI and computational methods that could ultimately result in reidentification. Such instances would indicate unawareness of misuse and accountability (Shahriari and Shahriari 2017). Therefore, our team decided to move to encoding key PHI and implemented explicitly stated analyses through Northwestern IRB, detailed below.

### Privacy and consent

This archive is a collection of internal documents from the nascent beginnings of the HGP to the modern genomic initiatives led by NHGRI. Unlike other scientific or bibliographic databases, the NHGRI archive houses documents created as a byproduct of knowledge production, including correspondences, emails, memos and notes from the leadership, administration, and employees of NIH and others involved in the HGP.^1^ Therefore, the archive’s contents capture individuals in varying degrees of reputation and prominence, from Francis Collins, former NHGRI Director (1993–2008) to staff and representatives from external organizations.

Individuals who worked on the HGP and subsequent initiatives have neither been approached nor agreed to have their email conversations or handwritten notes analyzed using AI and machine learning tools.^2^ Even if all data is fully de-identified and there is no risk of harm, we are analyzing user data without *explicit* consent. This could be viewed as undermining subjects’ autonomy and a harm in and of itself. Furthermore, the dataset includes both NIH and non-NIH employees. The NIH, as a federal agency under the Department of Health and Human Services, has explicit legal guidelines about the data that is kept during employment. As a government entity, the NIH has the responsibility to archive and preserve any and all documents deemed crucial. As part of being employed by the NIH, all NIH-affiliated individuals have consented to these recordkeeping procedures. However, the same cannot be said for involved individuals who were not employed by the NIH. For example, individuals in other academic institutions, private companies, and in other nation-states may have different recordkeeping policies, cultural norms, and social expectations about what is reasonable to be kept long term. By studying the documents created by these individuals without their consent, we may be infringing on their privacy and autonomy (insofar as they have no control over what happens to their information).

We do not have consent from all of those whose data is in the archive, nor can we acquire consent for the following reasons:

- The time period covered by the archive spans the early 1990s to the late 2000s; many individuals mentioned in the database have since retired, are deceased, or are otherwise unreachable.
- Individuals captured in the NHGRI archive include both the NIH employees and scientists from external organizations and foreign countries. Neither the NHGRI nor the NIH have access to the most recent contact information of all of these individuals.
- The NHGRI archive consists of both public (e.g. published papers, Congressional bills) and private (e.g. email conversations, informal drafts) documents. Therefore, it is not always possible to ascertain which individuals mentioned in a document would have to provide consent, and which individuals would not require consent (e.g. because data is already in the public domain).

After engaging with the data owners, machine learning experts, and data librarians, we decided on two acceptable scenarios:

- Conduct de-identification and declare the NHGRI archive de-identified.
- Detect and encode protected private information with a key and engage with Northwestern IRB to render it *encoded data*.

Our team opted for the latter solution. As described earlier, many remnants of data outside traditional PHI schemes can reidentify individuals in a de-identified dataset. Therefore, claiming a dataset to be fully de-identified would have downstream consequences: If considered fully de-identified, a dataset could be deemed appropriate for publication and sharing. Furthermore, the nature of the NHGRI archive makes it more difficult than other datasets to fully de-identify: The multi-modal and heterogeneous (text and image) data, handwriting, unstructured layouts, and informal language all make the full de-identification of the NHGRI archive a daunting task, if at all possible. By choosing to engage with IRB to render this data “encoded,” we acknowledged some of the aforementioned challenges, and proposed addressing them using institutional safeguards.

We employed several measures to minimize the risk of confidentiality breach. These included encoding possible names, physical addresses, social security numbers, credit card numbers, and email addresses from the NHGRI archive. Then, we developed in-house models to isolate and remove handwriting from all digitized documents in the archive. Because there are select documented instances of handwritten names, credit card numbers, and email addresses, handwriting is a source of PHI. However, as noted, traditional OCR engines like Tesseract cannot recognize handwritten characters, escaping de-identification efforts. By isolating handwritten sections, we can both remove handwritten pieces as well as send these to a separate pipeline dedicated to handwriting (Li et al. 2022). Although we have confirmed the technical possibility of separate processing, we currently abstain from it due to the challenging time requirements for reviewing the extracted metadata to ensure that no sensitive information will be released. Currently it remains unclear how much information is lost by removing handwritten notes. We estimate that around a third of the documents contain some form of handwriting (e.g. fully handwritten or containing other markings), which could add information beyond the printed content of these documents.

Furthermore, we fine-tuned existing named entity recognition models to include more entity categories. In many de-identification methods, either existing entity recognition datasets (Balasuriya et al. 2009; Sang and De Meulder 2003) are used or a major portion of the dataset-to-be-de-identified are labeled for fine-tuning. However, using these methods has several major challenges: The text in existing datasets are out-of-domain and do not appropriately fit the language in the dataset of interest; labeling parts of the dataset for fine-tuning introduces risks of exposing the very individuals we wish to de-identify during the annotation cycle; and, labeling datasets for fine-tuning is unsustainable and highly manual.

Therefore, we explored the use of synthetic entities, generated by rules and LLMs, to help fine-tune a pre-trained model. Recapitulating concerns echoed in the community about collapse of pre-trained models (Shumailov et al. 2023) and algorithm bias (Yu et al. 2023), we found that fine-tuning of entity detection models on in-domain NHGRI text was essential to reliably detect entities within the archive.

As a final step to ensure the security and privacy of the data, we placed it in an isolated and encrypted workstation to minimize exposure to malicious agents. Typically, use of AI approaches requires the use of powerful cloud based computing platforms. However, we avoided risks posed by the use of cloud resources by ensuring that this isolated workstation has enough computing power (GPUs, RAM, storage) to train state-of-the-art models without the need for cloud services.

### Ensuring maximum data security

The NHGRI History of the Human Genome Project and Genomics archive resides outside of NHGRI’s Division of Intramural Research and the Extramural Research Program, which are among the two main branches through which the NIH supports research. This necessitates setting up hierarchical access levels and precautionary measures (e.g. Data Transfer Agreement, Non-Disclosure Agreement) to ensure maximum data security. For example, we collaboratively decided to limit access of researchers at Northwestern to around 20,000 documents that have been reviewed by NHGRI. In addition some of these precautionary measures might prevent full adherence to FAIR (Findable, Accessible, Interoperable, Reproducible) principles and require redefining some of these principles to fit the contingencies of our project. For example, metadata about the content of documents is being created. As this metadata could contain sensitive information it will only be accessible to other scholars after signing of a Data Usage Agreement. While strictly speaking, this decision is not in-line with FAIR principles, it is a reasonable tradeoff because it helps mitigate risks, ensure ethical compliance, and maintain data privacy standards while enabling valuable research outcomes.

### Responsibility and accountability

Similar to other collaborations between humans and AI, responsibilities and accountabilities are subject to diffusion. Since AI is trained by humans, human collaborators are essentially also responsible for AI mistakes during the de-identification process. This is an ongoing issue that cannot be resolved.

If methods used in this project are reused by other scholars without the adopted due diligence and risk mitigating measures in this project, or worse, by malicious actors, we will enter the gray zone of accountabilities. While members of this project cannot be held liable for others’ mistakes, negligent scholars or malicious actors can claim that they used available tools and data and should not be held to account either, thereby creating an accountability impasse. To mitigate these risks, when posting the code and methods, we will develop and publish a brief “expectation of use” document that explains how the developed methods and tools should be used and what consideration should be observed in future studies to prevent harm.

## Who is impacted by this project?

This AI-powered content extraction and subsequent computational analysis impact the individuals discussed, mentioned, and captured in the NHGRI archive. These individuals vary in their roles, ranging from the top leadership of various departments at the NIH to intramural and extramural scientists and staff.

One expected outcome of this project is to gain a data-driven understanding of the practical operation of the scientific mechanisms inside funding institutions. The NHGRI archive contains materials regarding conception, clearance, and approval of large projects inside a focused Institute. Using machine learning models, we can explore factors that drove decisions and investigate how nascent beginnings of large projects emerged. In so doing, we would be able to identify inefficiencies, biases, or other factors that impacted the project timeline and their final outcomes. This knowledge would be beneficial to future administrators and the broader program evaluation services inside funding institutions.

This work is the first computationally driven analysis of a large internal archive of a major scholarly funding agency. We anticipate that our implementation will serve as an example for other archivists and library administrators in funding agencies that wish to study their own materials. Therefore, the procedures and policies set in place in this study will affect not only users of this data, but potentially owners and users of other archives who follow our implementation. We have already garnered attention and interest from other large archives in the biomedical sciences who wish to enhance their data in similar ways—data that also captures individuals and may expose them to harm if studied without careful procedures in place.

## Lessons learned and future work

We recognize that there is a difference between our use of AI and LLMs, and the possible utilization of these tools enabled by our research and the resulting machine-readable representation of information of the archive. Further, we cannot rule out the possibility that someone would use the data for malicious purposes that are presently unforeseeable to us. Complementing technical solutions to de-identify individuals, we hence set up a process that integrates into the current regulatory scheme developed by the NIH and that restricts the scope of use and limits various kinds of analyses. A possible path is to explicitly state what analyses can be conducted on this corpus, and excluding the rest (e.g. the type of work that is legally and regulatorily allowed to pursue with provided data such as various types of global network analysis), and the information that they are allowed to disclose.

## Documentation

**Figure 2:**
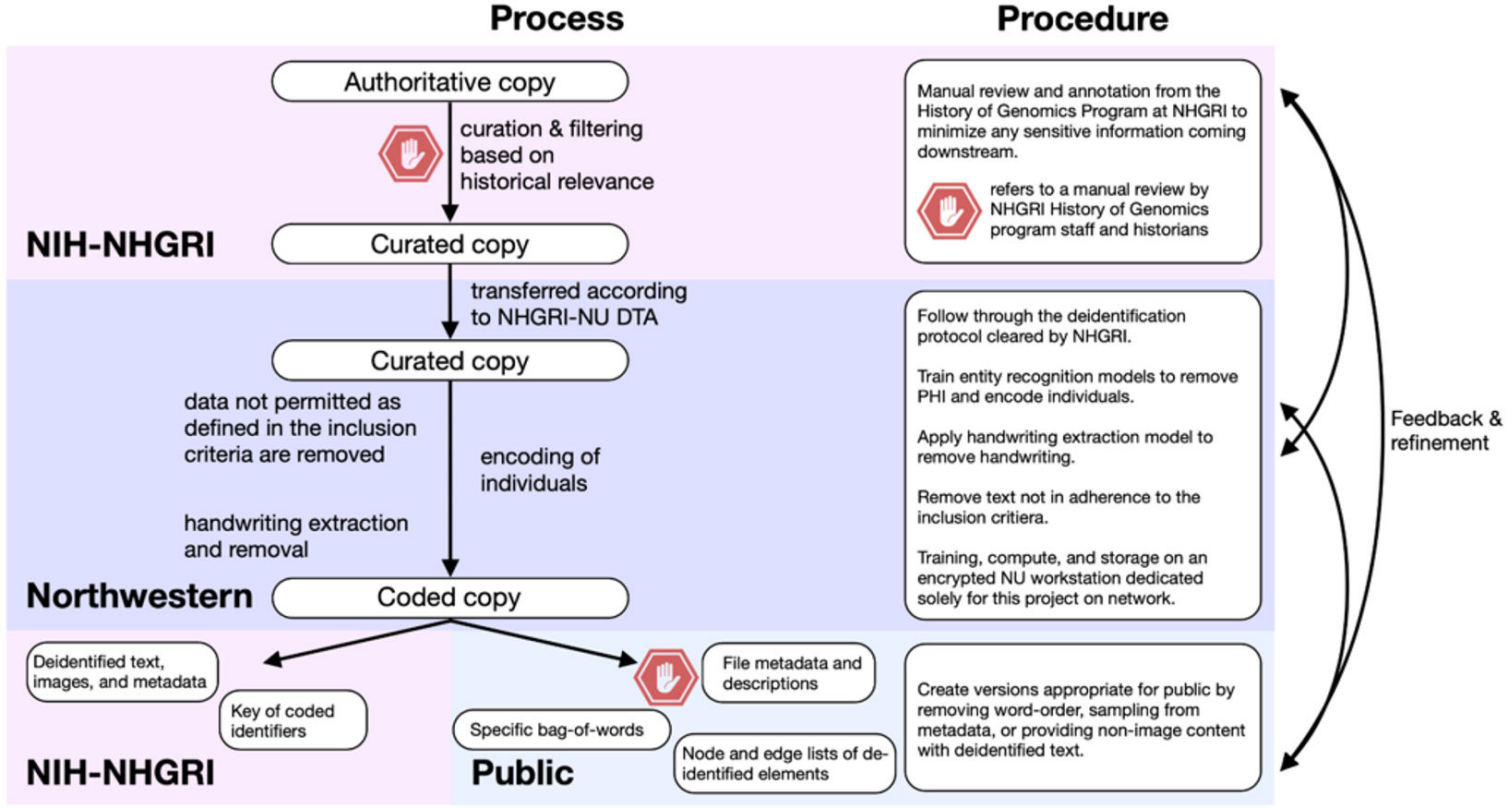
The IRB-approved process to handle the NHGRI archive data, including the transfer, handling, encoding, and usage of the documents.

Our consideration of the ethical aspects of the project have culminated in a submission to Northwestern University’s IRB prior to engaging in specific research-based analyses. The IRB categorized our study as low-risk, and therefore exempt. The workflow described in the IRB application is shown in figure 2.

## Data Availability

The content and metadata extracted from the archival materials of NHGRI are not available to be broadly shared due to the same ethical considerations discussed in this case study. The encoded metadata created from this project and the developed tools and models will be shared with a publication now in preparation. At present, access to the raw documents can be requested directly through the History of Genomics Program at the National Human Genome Research Institute.

## Footnotes

1 Our approach is inspired by principles and standards offered by the International Council on Archives (ICA) and the Open Archival Information System (OAIS) Reference Model on how to regulate the process of preserving and maintaining access to stored digital information over the long term.

2 Individuals who participated in the HGP, from the scientists who supported them and the institutions that funded them, are spread across many different institutions and nations. Their work and the corresponding material that came to be archived are therefore subject to different, and—at times—conflicting guidelines for future archival, analysis, and storage. Without consistent and explicit consent from all of the individuals mentioned in the archive, one could view this as undermining the subjects’ autonomy. We believe this to be an issue common in many other archival situations (such as Cold Spring Harbor) where the mentioned individual’s affiliation spans outside of the institution that is doing the archiving.
